# A 78.8–84 GHz Phase Locked Loop Synthesizer for a W-Band Frequency-Hopping FMCW Radar Transceiver in 65 nm CMOS

**DOI:** 10.3390/s22103626

**Published:** 2022-05-10

**Authors:** Van-Son Trinh, Hyohyun Nam, Jeong-Moon Song, Jung-Dong Park

**Affiliations:** Division of Electronics and Electrical Engineering, Dongguk University, Seoul 04620, Korea; trinhvanson92@dongguk.edu (V.-S.T.); kahn0217@dongguk.edu (H.N.); 2013111881@dongguk.edu (J.-M.S.)

**Keywords:** CMOS, frequency synthesizer, frequency hopping, FMCW radar, VCO, W-band

## Abstract

A W-band integer-N phase-locked loop (PLL) for a frequency hopping frequency modulation continuous wave (FMCW) radar is implemented in 65-nm CMOS technology. The cross-coupled voltage-controlled oscillator (VCO) was designed based on a systematic analysis of the VCO combined with its push-pull buffer to achieve high efficiency and high output power. To provide a frequency hopping functionality without any overhead in the implementation, the center frequency of the VCO is steeply controlled by the gate voltage of the buffer, which effectively modifies the susceptance of the VCO load. A stand-alone VCO with the proposed architecture is fabricated, and it achieves an output power of 13.5 dBm, a peak power efficiency of 9.6%, and a tuning range of 3.5%. The phase noise performance of the VCO is −92.6 dBc/Hz at 1-MHz and −106.1 dBc/Hz at 10 MHz offset. Consisting of a third-order loop filter and a divider chain with a total modulus of 48, the locking range of the implemented PLL with the cross-coupled VCO is recorded from 78.84 GHz to 84 GHz, and its phase noise is −85.2 dBc/Hz at 1-MHz offset.

## 1. Introduction

During the recent decade, advances in nanoscale Complementary Metal-Oxide-Semiconductor (CMOS) technologies have boosted the maximum oscillation frequency (*f_max_*) of transistors far from the mm-wave range, spurring an explosion of high-frequency applications such as millimeter-wave line-of-sight (LOS) wireless communication link system in the E-band, automotive radars in 77 GHz, or 3D imaging systems [1,2,3,4,5,6,7,8]. For the frequency modulated continuous wave (FMCW) radar sensor applications, frequency hopping approaches have been introduced to avoid intentional interference and security threats such as spoofing and jamming attacks [9,10]. A frequency synthesizer is an important part of the frequency hopping FMCW radar that is required to achieve a wide tuning range, and it also requires a low phase noise to ensure desired detection sensitivity and high output power to ensure the proper operation.

Many frequency synthesizers with various architectures or designing techniques have recently been reported [11,12,13,14,15,16,17,18,19,20,21,22,23,24,25]. Most of them can be classified into three groups: frequency-multiplication-based and oscillator-based synthesizers, and the architecture utilizing both types. The first group, i.e., a frequency multiplier (FM), directly multiplies a reference clock (*CK_ref_*) at low frequencies to the millimeter-wave range regime [22,23,24,25]. The phase noise (PN) performance of an *n*-time FM is estimated by PN (*CK_ref_*)–20log(*n*), where *n* is the multiplication factor. Therefore, FMs typically can achieve better PN performance compared to the oscillator-based frequency synthesizers owing to the better PN performance of the low-frequency signal sources. However, the reported multiplication factor to date is limited since an FM design can face issues of degraded stability and low conversion gain from the higher multiplication factors, as it requires cascaded multiple stages of the frequency multipliers. Moreover, an FM-based synthesizer still requires a high-performance signal source. By contrast, the oscillator-based frequency synthesizer normally requires a much lower *CK_ref_* that can be directly supported by low-cost crystal oscillators.

Due to environmental variables such as ambient temperature or supply voltage changes, an on-chip oscillator can face phase drift over time. There are two popular mechanisms used to lock the phase of an oscillator into a specific reference clock. One is a phase-locked loop (PLL) and another is injection locking. Each locking technique has its advantages and disadvantages. The injection-locked (IL) oscillators typically give better phase noises. However, the IL architectures are normally based on frequency multiplication which provides only low division ratios and requires high-frequency reference clocks [26]. Moreover, the injection pulling issues and the narrow locking range are widely conceived drawbacks of the IL oscillators [19]. The PLLs, by contrast, can provide wideband locking ranges, large division ratios that can support a low-frequency reference clock, and more stable operation [19]. The mixed architecture with PLL and FM can be built from a phase-locked oscillator at a low frequency, and then the output of the oscillator is multiplied in the frequency domain to attain the W-band signal [16,18]. In this mixed architecture, the divider chain design for the PLL is relaxed with a lower input frequency and the VCO can provide a better PN due to the higher quality factors of passive devices implemented at lower frequencies. Nevertheless, the low output power and the unwanted harmonics at the output of the FM are the challenges that a mixed frequency synthesizer may face.

**Figure 1:**
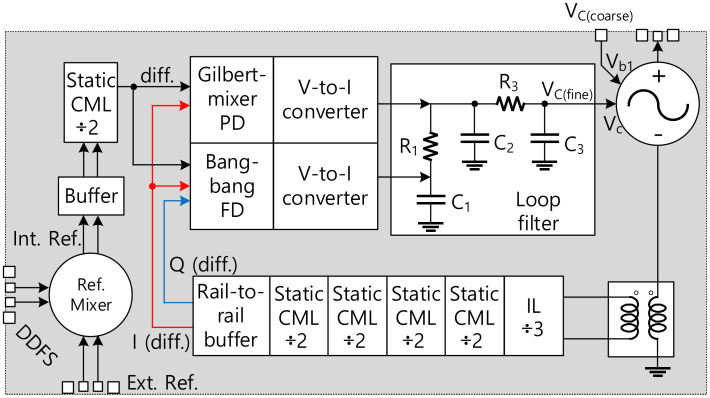
Block diagram of the implemented W-band PLL.

In this paper, we present a wideband W-band integer-N PLL using cross-coupled VCO specializing in FMCW radar applications, as illustrated by the block diagram in Figure 1. For an agile frequency control, the internal reference clock for the PLL is generated by mixing the external clock with the direct digital frequency synthesizer (DDFS) signal. An injection locking divider is employed at the first stage of the divider chain to deal with the high-frequency signal at W-band. The inductive feedback technique is applied for the divide-by-three IL divider to widen the locking range as presented in our previous work [27]. The fabricated PLL was measured and achieved a high output power and a good PN merit compared with other recently reported CMOS PLLs.

This paper is organized as follows. Section 2 presents the design and analysis of the cross-coupled VCO combined with an output buffer. The design of the PLL including the divider chain, phase frequency detector, and loop filter is described in Section 3. Section 4 discusses implementation and measurement, and is followed by a conclusion in Section 5.

## 2. W-Band Cross-Coupled VCO Design

**Figure 2:**
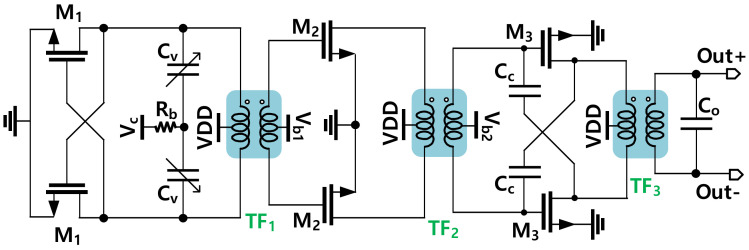
Schematic of the cross-coupled VCO with two buffer stages.

The whole schematic of the cross-coupled VCO with an output buffer is shown in Figure 2. Since the PLL output power is aimed at the milliwatt level for driving the transmitter power amplifier as well as supporting the locking operation of the divider chain, the output of the VCO is connected to a two-stage push-pull buffer to further boost its output power. Thus, it is important to co-design the push-push buffer with the VCO to achieve high efficiency.

### 2.1. VCO Configuration

The cross-coupled structure is a good choice for a simple, compact, and high-power VCO design. Moreover, with the use of a transformer at the output side, the VCO can naturally be connected to a push-pull amplifier to further boost its output signal. The output of the push-pull buffer can be changed from the single-end to differential configuration without incurring any additional passive element in driving the W-band Divide-by-Three Injection Locked Frequency Divider.

### 2.2. Cross-Coupled Pair Design Considerations

**Figure 3:**
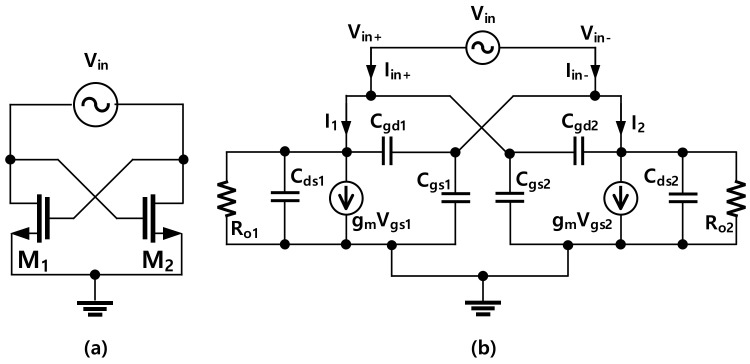
(**a**) A cross-coupled pair of NMOS with a port; (**b**) the simplified small-signal model of the circuit in (**a**).

In low-frequency regions, cross-coupled pairs of transistors (CCPs) commonly use ac-coupling capacitors in cross-feedback paths to bias the gate of the transistors separately from the supply voltage [28]. However, as the frequency becomes higher, a reliable ac-coupling capacitor becomes large compared with the transistor size. In addition, an ac coupling capacitor commonly possesses a low-quality factor. Thus, the gates of the transistors are normally connected directly to their counterpart’s drains as shown in Figure 3a. The schematic of the CCP using a simplified small-signal model of the NMOS with the output port is depicted in Figure 3b. In this model, if the output resistance (*R_o1_* and *R_o2_*) of the coupled pair is ignored and the circuit is symmetric, the input admittance of the coupled pair can be calculated as

(1) $$Y_{in}=\frac{I_{in}^{}}{\left(V_{in}^{+}-V_{in}^{-}\right)}=-\frac{1}{2}g_{m}+\frac{1}{2}\omega \left(4C_{gd}+C_{ds}+C_{gs}\right)$$

With this observation, the capacitance (*C_ccp_*) and the negative conductance (*G_ccp_*) of the CCP can be extracted from the input admittance as

(2) Cccp=Im{Yin}ω;Gccp=−Re{Yin}

**Figure 4:**
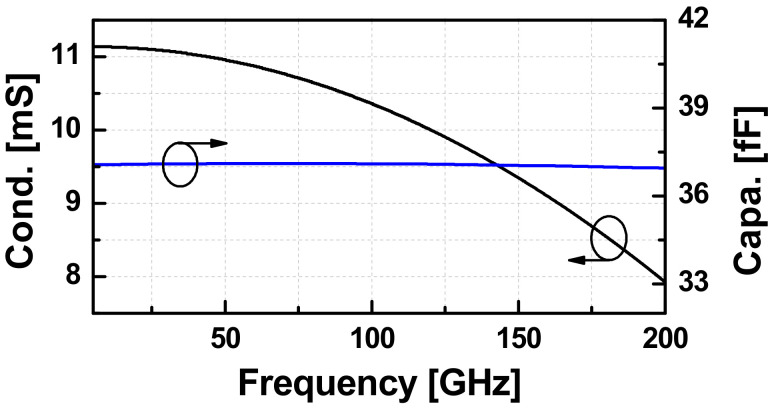
The small-signal extracted capacitance (*C_ccp_*) and negative conductance (*G_ccp_*) of a CCP with an NMOS the size of 32 μm versus frequency.

**Figure 5:**
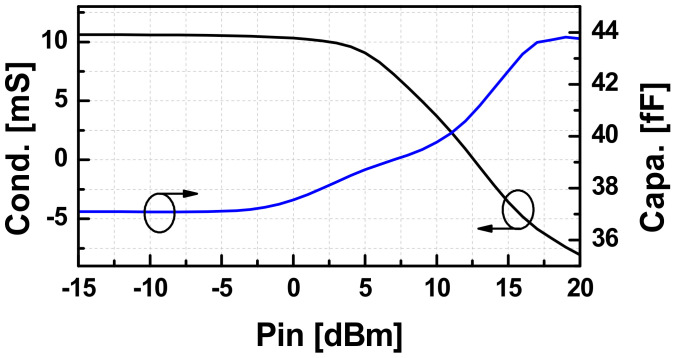
The HBSP-extracted effective capacitance (*C_ccp_*) and negative conductance (*G_ccp_*) of a CCP with an NMOS the size of 32 μm versus applied input power (*P_in_*) from the 50Ω-port.

Using (2), we extracted effective *C*_ccp_ and *G*_ccp_ of a CCP with the transistor size of 32 μm using the layout-extraction of the NMOS as depicted in Figure 4. Up to W-band, the model still works well when the extracted capacitance is almost constant and the negative conductance is slightly reduced as the frequency goes up. If the CCP of NMOS is connected to an inductive circuit such that the external loss is smaller than the active energy generated by *G_ccp_*, then the whole circuit will start to oscillate under a presence of a trigger. The swing will keep growing until *G_ccp_* is compressed to be the same as that of the loss. Figure 5 demonstrates the large-signal values of *C_ccp_* and *G_ccp_* extracted from the harmonic-balance s-parameter (HBSP) simulation running at 82 GHz. The *P*_in_ in the graph is the power generated from the 50 Ω port.

It can be observed that *G_ccp_* degrades quickly when *P_in_* is larger than 4 dBm, and the device loses its active characteristic when the signal is stronger than 12.5 dBm. The capacitance of the pair increases as the oscillation signal is stronger. Hence, the oscillation frequency is slightly reduced compared to the calculated value based on the extracted *C_ccp_* under the small-signal regime.

### 2.3. Variable Capacitor Design

**Figure 6:**
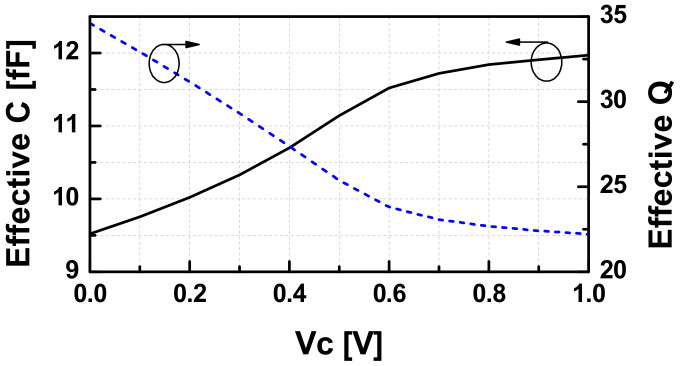
The input admittance of the buffer versus gate voltage bias.

There are three types of variable capacitors available: thin gate NMOS, thin gate PMOS, and thick gate NMOS in the used 65 nm CMOS process. Among them, the thin gate PMOS capacitor has the highest Q-factor with the same fractional capacitance tuning range. Figure 6 depicts the effective capacitance and the Q-factor of the variable capacitor used in the designed VCO.

### 2.4. VCO Buffer Design with a Coarse Control Knob

Push-pull amplifiers have been widely used in PA design due to their compactness, high efficiency, and high-power performances [29,30]. Since the load of the first buffer stage also affects the tuning range of the VCO, the active device size of the first stage should be restricted to keep a reasonable tuning range of the VCO. Hence, two more push-pull amplifying stages were employed to further boost the output power of the VCO to keep the output power large enough to drive the W-band divide-by-three injection-locked frequency divider. The two-stage buffer structure also enhances the isolation between the CCP and output of the VCO, mitigating the pulling effect of the CCP core from the load variation. It is well-conceived that the differential pair of active devices constructed by two common source amplifiers is conditionally unstable due to the gate-to-drain parasitic capacitor *C_gd_* of the transistors [29]. Regarding the first buffer stage, it is possible to allow it to be conditionally unstable with a stability factor smaller than unity since its input resistance generated by the cross-coupled pair is inherently negative. To stabilize a differential transistor pair of the second buffer stage, we employed the capacitive neutralization technique by adding cross-connected capacitors between the gate and the drain of the pair transistor, which is close to the *C_gd_* of the transistors [29].

**Figure 7:**
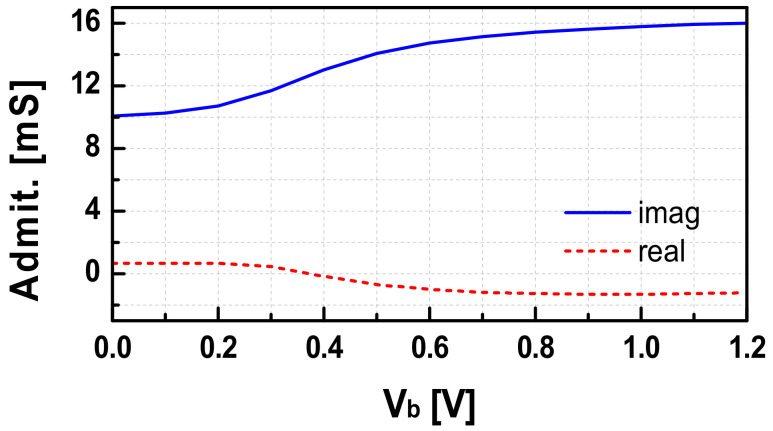
The input admittance of the buffer versus gate voltage bias (*V_b1_*).

Theoretically, if *C_gd_* is neglected, the input impedance observed from the buffer to the load is only the gate capacitance with its parasitic resistance. Therefore, the expected input admittance of the buffer should be a capacitive susceptance in parallel with a conductance. These values were extracted at 82 GHz with various bias values of *V_b1_*, as shown in Figure 7. As observed, the input susceptance of the first buffer varies largely (~60%) depending on the bias voltage (*V_b1_*) of the gate. Therefore, we can effectively tune the oscillation frequency of the VCO by controlling the gate bias voltage (*V_b1_*) of the first stage of the buffer. In the overall synthesizer, *V_b1_* is used as the coarse tuning knob (*V_C(coarse)_*) to change the output frequency of the PLL while the control voltage of the variable capacitor, *V_c_*, in the VCO is used as a fine-tuning knob (*V_C(fine)_*) to tune a proper *K_VCO_* value in the PLL locking operation. In this way, the frequency hopping operation of the designed PLL could be effectively achieved without any implementation overhead.

### 2.5. VCO Design

**Figure 8:**
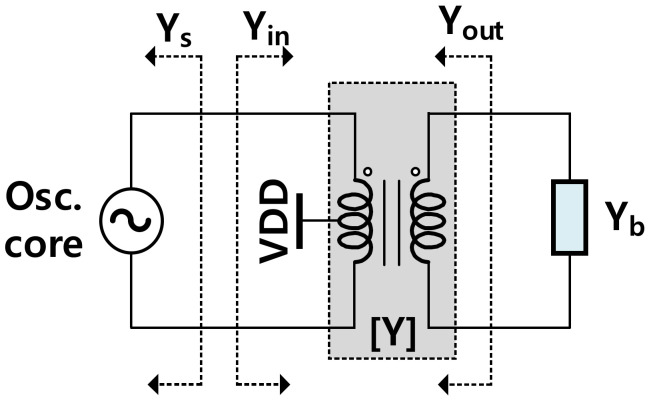
The schematic circuit of the VCO using a transformer at the output.

We employed a transformer as the interconnection between the VCO core and buffer as illustrated in Figure 8. Herein, the VCO core including the CCP and the variable capacitors are modeled as *Y*_s_ = *G*_s_ + *jB*_s_; the transformer can be generally modeled by a two-port *Y*-parameters matrix; the buffer is characterized by *Y*_b_ = *G*_b_ + *jB*_b_. The input admittance, *Y*_in_, and the output admittance, *Y*_out_ can be calculated as

(3) $$Y_{in}=G_{in}+jB_{in}=y_{11}-\frac{y_{12}y_{21}}{y_{22}+Y_{b}}$$

(4) $$Y_{out}=G_{out}+jB_{out}=y_{22}-\frac{y_{12}y_{21}}{y_{11}+Y_{s}}$$

where *y*_11_, *y*_12_, *y*_21_, and *y*_22_ are elements of the transformer’s *Y* matrix. The VCO will oscillate at the frequency where the inductive susceptance of *Y*_in_ is equal to the capacitive susceptance of *Y*_s_ [31], i.e.,

(5) $$B_{in}(\omega_{osc})=-B_{s}(\omega_{osc})$$

For the VCO to oscillate, the negative conductance generated by the CCP has to be larger than the total conductance of *y*_in_ and the variable capacitor, i.e.,

(6) $$\left|G_{s}\right|>G_{in}\Leftrightarrow \left|G_{ccp}\right|>G_{\mathrm{var}\_cap}+G_{in}$$

Under an appearance of a trigger, the VCO will start to oscillate. The swing will grow until the effective negative conductance of the CCP is equal to the loss from the load. At this operating signal level, the exchange between electric and magnetic energy is balanced; i.e., *eff*(*Y_s_*) = −*eff*(*Y_in_*) at the operating point, and one can verify that under this condition, we also have *eff*(*Y_out_*) = −*eff*(*Y_b_*).

Conjugate matchings at both sides of the transformer should be obtained to maximize its efficiency. Thus, we want to set the admittances as below:

(7) $$G_{s}=G_{in};G_{b}=G_{out}$$

(8) $$B_{s}=-B_{in};B_{b}=-B_{out}$$

The local optimization for the impedance matching level concerning *B*_s_ and *B*_b_ is attainted with conditions in (8). Interestingly, the input matching formula in (8) is also the condition for the oscillation frequency in (5).

The low-frequency lumped model of a transformer consists of five parameters including *L*_1_, *R*_1_, *L*_2_, *R*_2,_ and *M,* which characterize the two winding inductors and their mutual inductance, respectively [32]. The coupling coefficient is defined as *k* = *M*/(*L*_1_*L*_1_)^1/2^. Two quality factors of the two winding inductors are calculated as *Q*_1_ = *ωL*_1_/*R*_1_ and *Q*_2_ = *ωL*_2_/*R*_2_. The input and output matching susceptance is calculated by [29].

(9) $$B_{s\_opt}=\frac{1}{\omega L_{1}}\times \frac{Q_{1}^{2}}{1+Q_{1}^{2}+k^{2}Q_{1}Q_{2}}$$

(10) $$B_{b\_opt}=\frac{1}{\omega L_{2}}\times \frac{Q_{2}^{2}}{1+Q_{2}^{2}+k^{2}Q_{1}Q_{2}}$$

Formulae in (9) and(10) imply that it is better to design a small transformer for the interconnection between the CCP and the buffer. A smaller primary winding inductance of the transformer can support a larger size of the transistor CCP as well as the variable capacitor. Similarly, at the transformer’s secondary side, it is possible to match a bigger buffer, which can generate a higher output power. However, a small transformer may result in a low coupling factor between the two coils, which degrades the transformer efficiency, assessed by *k*^2^*Q*_1_*Q*_2_ [32]. Moreover, the accuracy of simulation can deteriorate when the routing layout becomes considerable compared to the modeled transformer.

**Figure 9:**
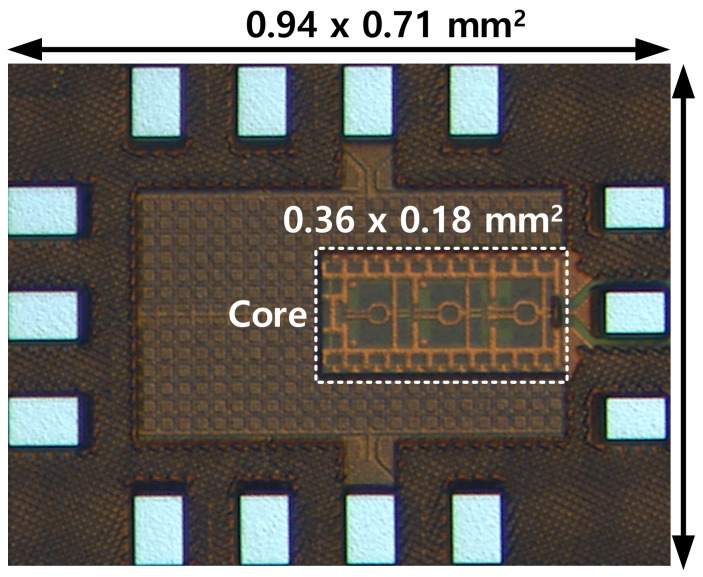
A photograph of the standalone VCO fabricated in 65-nm CMOS.

The VCO in the PLL is implemented with the element parameters given in Table 1. A stand-alone (SA) version of VCO having the same structure with a single-ended output is implemented with a set of circuit parameters given in the same table. The SA VCO is designed to support a full band measurement, and it is optimized in terms of output power performance with a larger transistor in the second push-pull amplifier. Meanwhile, the VCO in the PLL was designed with a larger tuning range to support the locking operation of the PLL. A photograph of the SA VCO chip fabricated on 65-nm CMOS is shown in Figure 9. The chip size of the full SA VCO including all DC and radio frequency (RF) pads is 0.667 mm^2^, and the core size is 0.065 mm^2^.

## 3. Integer-N Phase-Locked Loop Design

### 3.1. Divider Chain

**Figure 10:**
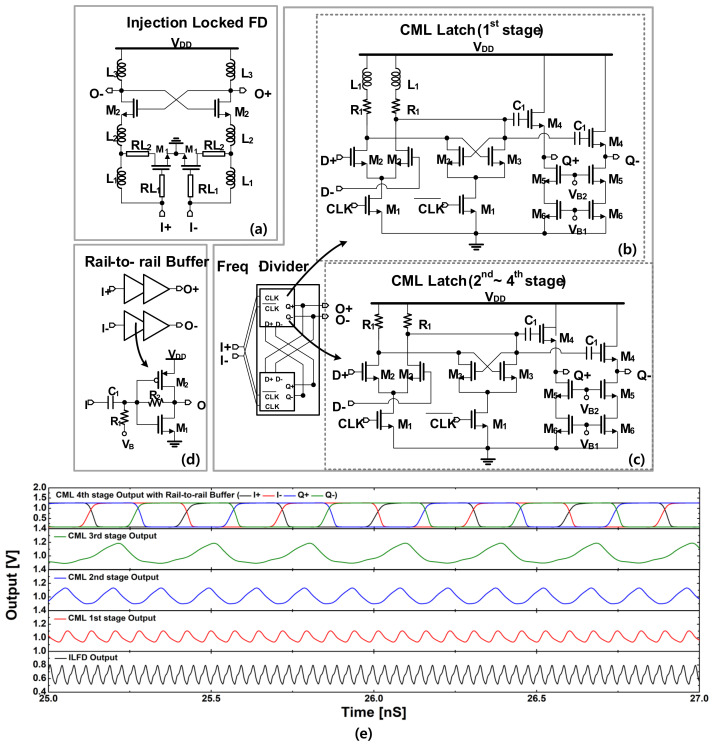
Schematic of the divider chain: (**a**) the injection-locked FD; (**b**) the first CML FD; (**c**) the second, third, and the fourth CML FD; (**d**) rail-to-rail buffer; (**e**) A simulation result of the divider chain.

We employed an integer-N PLL considering its simplicity and robustness. To achieve a fast settling time, the reference frequency was chosen to be relatively high by setting the modulus N = 48. The divider chain is composed of five stages to achieve the desired modulus. Different divider structures were employed along the chain to achieve proper frequency division at different frequency regimes. The schematic of the divider chain is depicted in Figure 10. There are three divider topologies popularly used in the millimeter-wave and microwave regime, which include injection-locked (IL) dividers, Miller dividers, and static current mode logic (CML) dividers. Among them, the IL topology achieves the highest frequency operation due to its injection locking mechanism [33]. Hence, the first stage is realized as an injection locking divide-by-three divider (ILFD) to deal with a high-frequency input at the W-band generated by the VCO. An inductive feedback network was employed for transistors *M*_1_ of the ILFD as shown in Figure 10a to enhance its locking range. This technique was demonstrated to improve the fractional bandwidth of the ILFD up to approximately 11% [27]. Since the ILFD required a specific level of input signal for a proper locking operation, which is simulated to be around 0-dBm, the VCO output was designed to achieve at least this signal level in the worst condition, with a safety margin considering the process variation.

Working at a lower frequency, the four remaining stages were realized as static CML dividers (CML-FDs) to achieve a wider band operation, as shown in Figure 10b,c. Nevertheless, the operation frequency of the first CML divider stage is still relatively high, thus additional inductors are connected in series to the load of the first CML-FD to increase its output voltage swing as shown in Figure 10b. Each CML-FD includes a source follower (SF) stage to increase its driving capability to the next stage. The output of the divider chain is finally connected to two stages of inverter-type buffers to generate the rail-to-rail output driver for the FD and PD, as shown in Figure 10d. A simulation result of the divider chain is shown in Figure 10e when a W-band input of 0-dBm is introduced to the divide-by-three ILFD, and it shows all the divider stages functioned normally with output voltage waveforms shaped as expected.

### 3.2. PD, FD, Loop Filter, and the Reference Clock

**Figure 11:**
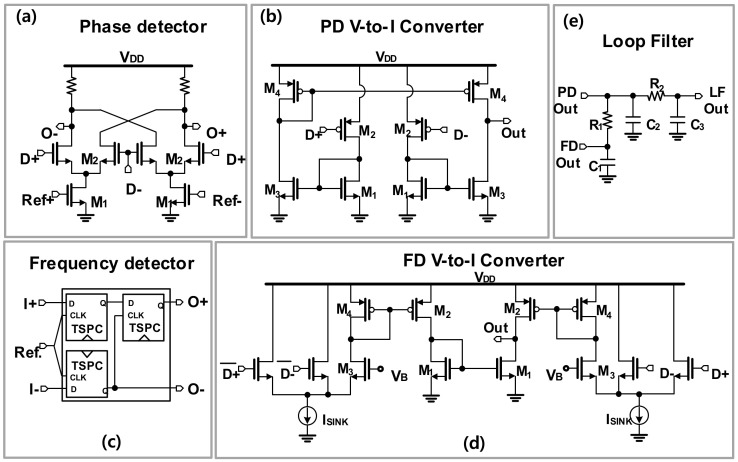
Schematics of (**a**) the phase detector; (**b**) the V-to-I converter for the PD; (**c**) frequency detector; (**d**) V-to-I converter for the FD, and (**e**) the loop filter.

The schematics and the simulation results of the PD, FD, and the loop filter are presented in Figure 11. The PD is implemented using a Gilbert-mixer structure to achieve good compression of the reference spurs and mitigate the dead-zone problem [11,34,35]. When the loop is locked, the PD output contains an AC signal at twice the reference signal frequency and a DC component with the value functioning as the cosine of the phase difference between the two inputs [34]. It is noticed that the large reference frequency facilitates the loop filter in suppressing the AC component in the output of the PD. The phase difference between the two inputs of the PD is 90° in the locking condition. Then, the output voltage signal of the PD is converted to the current by the V-to-I converter to drive the loop filter to control VCO, as shown in Figure 1.

**Figure 12:**
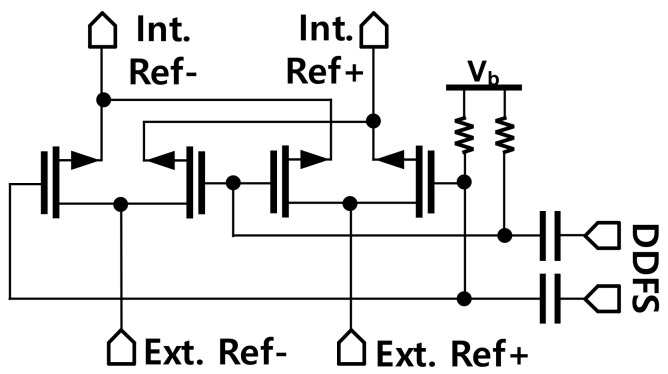
Schematic of the mixer to generate the internal reference frequency for the PLL.

The FD helps to expand the frequency locking range of the PLL, and it is realized with a typical bang-bang structure, as given in Figure 12c. In the designed FD, three high–speed D-type flip-flops are used to extract the frequency different values between the two inputs. The detailed operating principle of the FD can be found elsewhere [35]. When the frequency is locked, the bang-bang FD automatically switches off to avoid any disturbance to the VCO. The associated V-to-I converter of the FD is shown in Figure 12d. It is designed to have a larger pumping current compared to that of the PD to ensure the dominance of the FD during the frequency acquisition period.

The loop filter is designed based on the typical third-order filter as shown in Figure 12e. The loop bandwidth was chosen to be higher than 10 MHz to reduce the settling time of the locking operation, which is an important factor in the FMCW radar system where the reference frequency of the PLL is swept linearly in a short stairstep manner.

**Figure 13:**
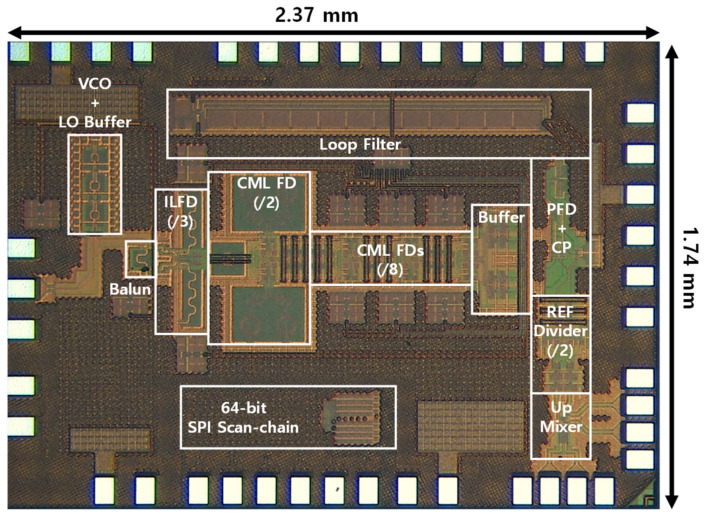
A microphotograph of the proposed PLL.

The internal reference frequency (*F_int_ref_*) for the PLL is generated by mixing the external reference signal with the frequency swept signal generated from the Direct Digital Frequency Synthesis (DDFS) using a double-balanced passive mixer as depicted in Figure 12. The internal reference clock is then amplified by two stages of the inverter-type amplifier with the designed bandwidth to reject the image from the mixer. The PLL was implemented in 65-nm CMOS technology and a microphotograph of the PLL is presented in Figure 13. The whole PLL including pads occupies an area of 2.37 × 1.74 mm^2^.

## 4. Measurement Results

### 4.1. Stand-Alone VCO Measurements

**Figure 14:**
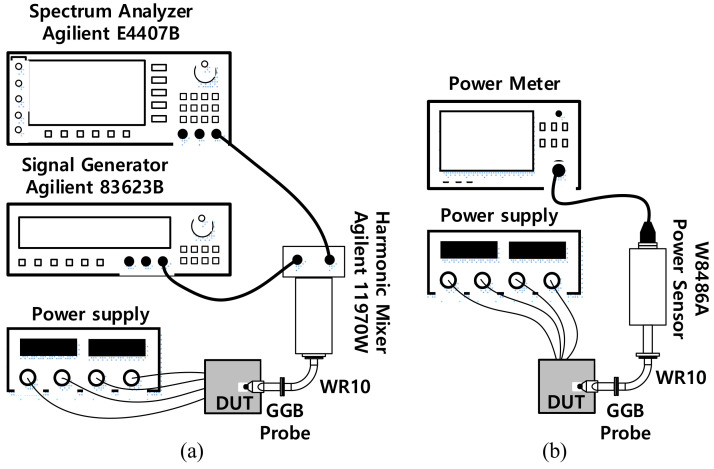
Measurement setups for the SA VCO: (**a**) frequency and phase noise (**b**) output power.

**Figure 15:**
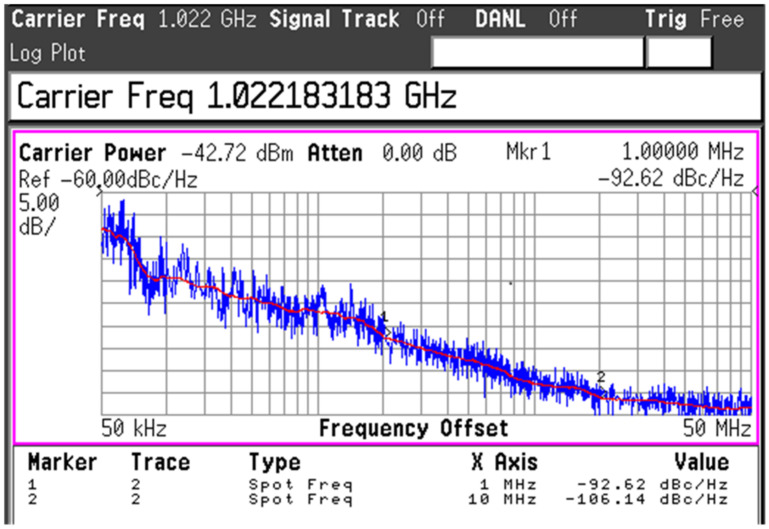
The measured phase noise of the stand-alone VCO.

**Figure 16:**
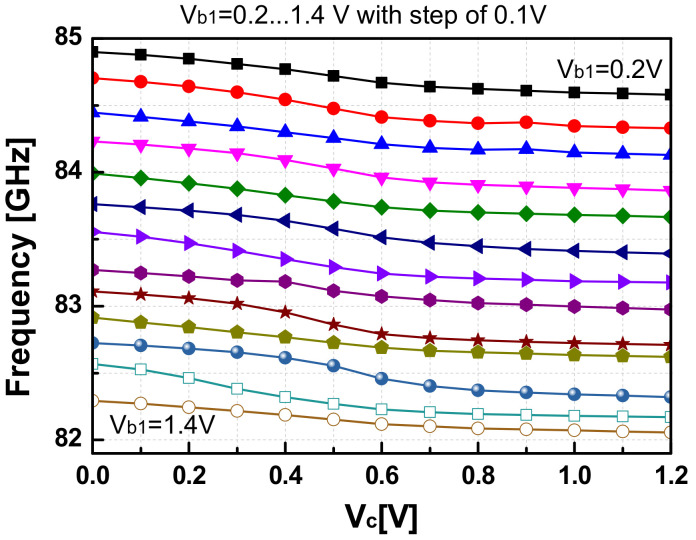
The measured oscillation frequency of the SA VCO as a function of *V_c_* = V_c(fine)_.

The setups to measure the oscillation frequency (*F*_osc_), phase noise (*PN*), and the output power (*P*_out_) of the VCO are demonstrated in Figure 14. An 18th harmonic mixer (Agilent 11970W) was used to measure the spectrum of the W-band signal from the VCO by down-converting the VCO output with a LO signal from the signal generator (Agilent 83623B). The intermediate frequency signal (IF) was then input into a spectrum analyzer (E4407B) to measure the frequency and PN performance. The conversion loss of the harmonic mixer and other cable connections was estimated to be around 48-dB or even higher (~50-dB) depending on IF frequency, as estimated in [11]. Figure 15 shows the PN performance of the VCO when *V*_b1_ = *V*_b2_ = 0.7 V, which was read to be −92.6 dBc/Hz and −106.1 dBc/Hz at 1-MHz and 10-MHz, respectively. The phase noise of the signal generator was measured to be −128.3 dBc/Hz at 1-MHz offset and −144.4 dBc/Hz at 10-MHz offset. This means the PNs of the LO signal after being multiplied 18 times are calculated by −103.2 dBc/Hz at 1-MHz offset and −119.3 dBc/Hz at 10 MHz. Thus, the PNs of the measurement setup were still more than 10-dB smaller compared with the measured PNs of the VCO signal at corresponding frequency offsets, which justifies the PN measurement results from the IF of the harmonic mixer. The measured *F*_osc_ of the VCO is shown in Figure 16. When the varactor control voltage (*V_c_*) varies from 0-V to 1.2-V and the bias tuning voltage (*V_b1_*) varies from 0.2-V to 1.4-V, the oscillation frequency of the VCO changes from 82-GHz to 84.9-GHz.

**Figure 17:**
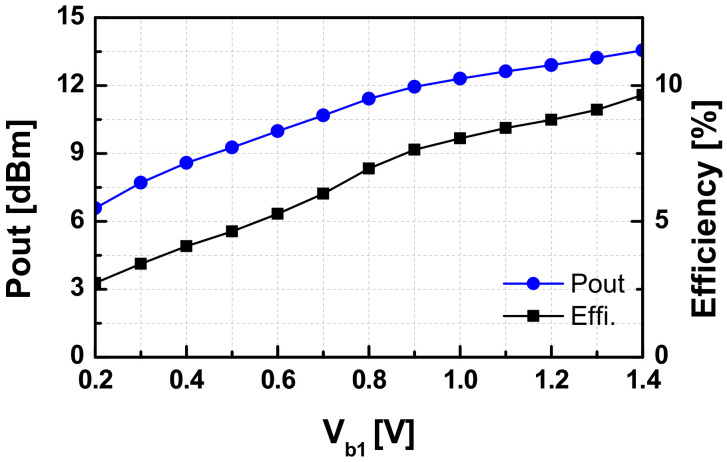
The measured output power and efficiency of the VCO versus bias voltage *V_b1_* = *V_C(coarse)_.*

The output power was measured by a W-band power sensor (W8486A) combined with a power meter. The external losses of the probe and the waveguide connections were calibrated from the read output power of the VCO. In the measurement, the output power of the VCO was almost unchanged, as *Vc* varies. However, it depends on the value of *V_b1_* as shown in Figure 17. The calculated efficiency from those values is shown in the same figure as well. The measured results of the SA VCO were summarized in Table 2 in comparison with other state-of-art VCOs reported recently [36,37,38,39,40,41,42,43,44,45]. The designed VCO in this work achieved superior output power and high efficiency among W-band CMOS VCOs owing to the efficient co-design of the output buffer.

### 4.2. PLL Measurements

**Figure 18:**
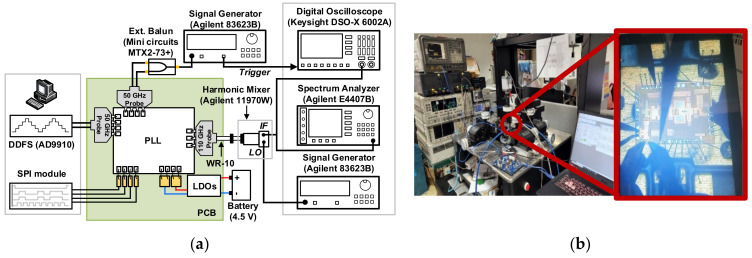
PLL measurement setup: (**a**) schematic diagram; (**b**) photo.

**Figure 19:**
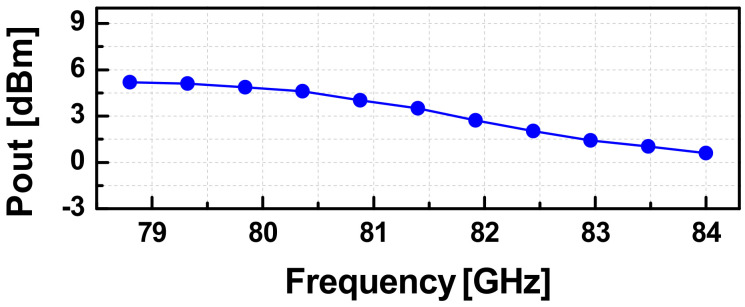
The measured output power of the PLL versus frequency.

**Figure 20:**
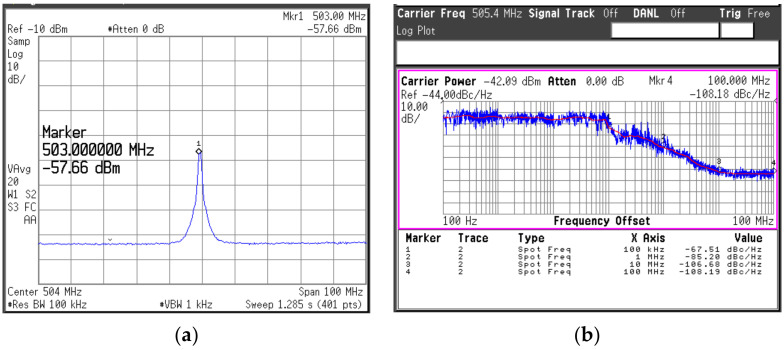
Measured output spectra of the PLL: (**a**) spectrum; (**b**) phase noise.

The measurement setup for the PLL is similar to that for the SA VCO, and it is shown in Figure 18. Herein, for typical measurements as an oscillator, the DDFS ports were injected by a continuous wave signal from a signal generator. Under a supply of 1.2-V, the PLL consumed a dc-power of 301.4 mW at the coarse tuning voltage of *V_b1_* = 0.2-V and 351.4-mW with *V_b1_* = 1.2-V. The whole locking range of the PLL was recorded from 78.84 to 84 GHz with the continuous tuning range of *V_b_*_1_ from 0.2 to 1.2 V. For a fixed value of *V_b_*_1_, the locking range of the PLL was measured to be ~400-MHz, and it is slightly reduced as *V_b_*_1_ increases. The measured output power of the PLL versus the operating frequency is given in Figure 19. The PLL generated an output power larger than 0.6 dBm over the whole locking band with a peak of 5.2-dBm at 78.8-GHz. A down-converted IF spectra of the PLL in the locking condition and its PN performance are shown in Figure 20a. The reference spur in this case was dominated by the high noise level of the harmonic mixer used in the measurement. Due to the absence of the reference spur in the measured IF, we can estimate that it should be smaller than the noise level, i.e., smaller than ~40 dBc compared to the main tone. The measured PNs at 1-kHz, 1-MHz, and 10-MHz were −67.5, −85.2, and −106.7 dBc/Hz, respectively, as shown in Figure 20b.

**Figure 21:**
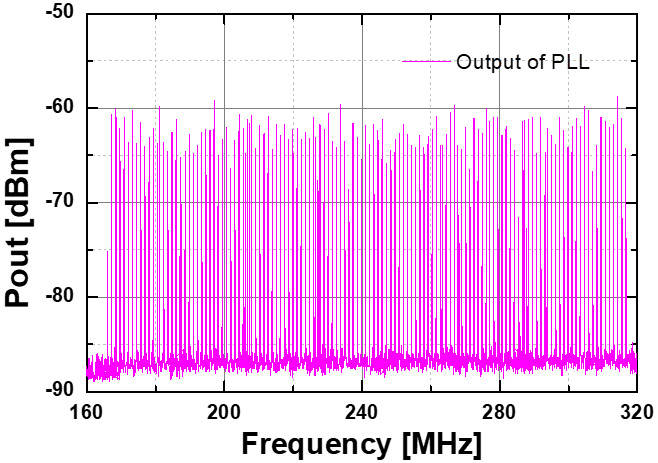
Measured down-converted output spectra of the frequency-modulated PLL with the modulation frequency *f_m_* = 1 MHz and coarse tuning bias voltage *V_C(coarse)_* = *V_b1_* = 0.66-V.

To verify the operation of the PLL for an FMCW radar transceiver, a DDFS was used to generate a sweeping signal with the frequency changed by step, which was injected into the DDFS port of the PLL. The PLL’s output was then down-converted to the IF band by using the harmonic mixer Agilent 11970W, which was finally captured on the spectrum analyzer (E4407B). Figure 21 shows the measured down-converted output spectrum of the PLL with a frequency-modulated signal with the linear frequency step of 1-MHz with *V_b1_* = 0.66-V. As can be observed, the output of the PLL can track the modulated signal of the DDFS port correspondingly.

The performance of the PLL is summarized in Table 3 in comparison with other recently reported CMOS and SiGe PLLs around the W-band.

## 5. Conclusions

We presented a W-band frequency synthesizer implemented with an integer-N phase-locked loop (PLL) with a fundamental VCO for a frequency hopping FMCW radar transceiver in 65-nm CMOS technology. The cross-coupled pair of active devices were used as the core of the VCO and a two-stage push-pull buffer was co-designed to achieve high power and efficiency. By controlling the gate voltage of the first stage of the VCO buffer, the output frequency could be effectively hopped without any implementation overhead. A standalone version of VCO with an enhanced output power was verified and it achieved an output power of 13.5 dBm, and the phase noise performance of −92.6 and −106.1 dBc/Hz at 1-MHz and 10-MHz offsets. Considering a fast settling time, the PLL utilized a modulus N = 48. The first divider stage was implemented with a divide-by-three injection-locked divider using inductive feedback to expand the locking range. The implemented W-band PLL achieved an output power of 5.8-dBm with PN of −67.5 and −85.2 dBc/Hz at 1-MHz and 10-MHz, respectively. The modern radar and wireless communication systems acquire higher performance frequency synthesizers in terms of phase-noise, output power, and tuning range to catch the trend of multiple target tracking in radars [46] or improve the inter-symbol interference (ISI) in communication systems when higher carriers are employed to support broadband data rate [47]. The presented W-band wideband PLL architecture is applicable to various CMOS transceivers in FMCW radar and 6G communication applications.

## Figures and Tables

**Table 1:** Circuit component parameters of the two VCOs.

Device	Size (in PLL)	Size (SA VCO)
M_1_	W = 32 μm; L = 65 nm	W = 32 μm; L = 65 nm
C_v_ (MOS CAP)	W = 7.2 μm; L = 0.5 μm	W = 5.4 μm; L = 0.5 μm
TF_1_	Din = 18 um	Din = 20 um
M_2_	W = 40 μm; L = 65 nm	W = 46μm; L = 65 nm
TF_2_	Din = 26 μm	Din = 22 μm with an additional inductor at the secondary side
M_3_	W = 80 μm; L = 65 nm	W = 140 μm; L = 65 nm
C_c_ (MOM CAP)	16 fF	34 fF
TF_3_	Din = 24 μm	Din = 22 μm
C_o_ (MOM CAP)	26 fF	28 fF

**Table 2:** Comparison of state-of-the-art VCOs around W-band.

Ref.	Structure	Process	VDD/ VCC	Freq. (GHz)	Tuning Range	Pout (dBm)	P_DC_ (mW)	Phase Noise (dBc/Hz)	DC-RF Eff. (%)	Area (mm^2^)	FOM
**This**	**Cross-coupled**	**65-nm CMOS**	**1.2 V**	**83.5**	**3.5**	**13.5**	**167–235**	**−92.6@1 MHz** −**106.1@** **10 MHz**	**9.6**	**0.065**	**−205.4**
[36]	Diff. Colpitts	90-nm CMOS	1.8 V	97.7	7.2	4.7	6–63	−80@ 1 MHz	5.5	0.063	−189.0
[37]	Coupled Oscillator	65-nm CMOS	1.2 V	105	9.5	4.5	54 ^(1)^	−92.8@ 1 MHz	5.2	0.228 ^(2)^	−204.4
[38]	Ring Oscillator	130-nm CMOS	1.5 V	104	NA	−2.7	28	−93.3@ 1 MHz	1.9	0.16 ^(2)^	NA
[39]	Push-Push	65-nm CMOS	1 V	81.5	14	−0.5	33	97.3 ^(5)^@ 1 MHz	2.7	0.046	202.3 ^(5)^
[40]	Pseudo-diff. ^(4)^	65-nm CMOS	1 V	77	14.5	6.2	190	−88@ 1 MHz	2.2	NA	−198.6
[41]	Cross-coupled	130-nm CMOS	1.8 V	91	0.5	4.5	46	−87@ 1 MHz	6.1	0.51 ^(2)^	−172.5
[42]	Diff. Colpitts	65-nm CMOS	1.8 V	98	8.7	−1	4–21.6	−90@ 1 MHz	3.7	0.18 ^(2)^	−193.3
[43]	Colpitts-based	130-nm SiGe	2.5 V	74	4.86	2 ^(3)^	65	−99.3@ 1 MHz	2.4	0.017 ^(6)^	−196.2
[44]	Self-feeding	130-nm SiGe	0.82–0.96 V	88	1 ^(7)^	7.5 ^(7)^	-	−106.9 ^(7)^@ 1 MHz	19.4 ^(7)^	0.028 ^(2)^	−186.2
[45]	Colpitts-based	130-nm SiGe	1.2–1.65 V	103.5	28	4.2	50	−108.7@ 1 MHz	5.3	0.16	−229.4

^(1)^ Power consumption of the core only, excluding the buffers. ^(2)^ Area including the pads. ^(3)^ Estimated using the transmitter and power amplifier measurement. ^(4)^ The fundamental VCO is at 38 GHz, then it feeds the signal to a doubler and a buffer. ^(5)^ Including buffer for fundamental oscillation signal. ^(6)^ Area of the core only (without buffer). ^(7)^ A varactor-less design with tuning supply voltage using an external bias tee $FOM=FN(\Delta f_{offset})-20\log\left(\frac{f_{0}}{\Delta f_{offset}}\times \frac{TR[\%]}{10}\right)+10\log\left(E[\%]\right)+P_{out}[dBm]$.

**Table 3:** Comparison of state-of-the-Art PLLs around W-band.

Ref.	Tech.	Frequency (GHz)	TR (%)	PN @1 MHz (dBc/Hz)	Reference Frequency	Reference Spur (dBc)	P_out_ (dBm)	P_diss_ (mW)	Division Ratio	Area (mm^2^)
**This**	**65-nm CMOS**	**78.84~84**	**6.4**	**−85.2**	**1.64–1.75 GHz**	**12.4**	**5.8**	**326.4**	**48**	**4.12**
[11]	90-nm CMOS	73.4~73.72	0.43	−92	2.34 GHz	2.5	−18.5	88	32	0.8
[12]	65-nm CMOS	95.1~96.5	1.5	−75.9	373 MHz	2	−26.7	43.7	256	0.7
[13]	65-nm CMOS	70~78	10.8	−83	70 MHz	0.3	−18.9	76	1024~1984	0.31
[14]	65-nm CMOS	96.8~108.5	11.5	−88	195 MHz	1	NA	14.1	512	0.39
[15]	65-nm CMOS	103~104.5	1.5	−80.4	406 MHz	2	−23.1	63	256	0.84
[16]	90-nm CMOS	76.2~89.1	15.6	−87.9	50 MHz	0.1	−2.9	62.4	512	1.3
[20]	65-nm CMOS	93.4~104.8	11.5	−85.75	100 MHz	NA	NA	57	NA	0.8755
[21]	65-nm CMOS	82.0~107.6	27	NA	125 MHz	NA	NA	35.5	NA	0.36
[17]	130-nm SiGe	86~92	6.7	−100	0.6~6 GHz	1.7~8.5	−3	1150	16	1.87
[18]	180-nm SiGe	90.9~101.4	10.9	−92	125 MHz	1	−11	140	256	1.9
[19]	130-nm SiGe	92.7~100.2	7.8	−102	3 GHz	20	3	469.3	64	0.93
